# Characterization of the post-prandial insulinemic response and low glycaemic index of a soy beverage

**DOI:** 10.1371/journal.pone.0182762

**Published:** 2017-08-09

**Authors:** Jose CE Serrano, Meritxell Martín-Gari, Anna Cassanye, Ana Belen Granado-Serrano, Manuel Portero-Otín

**Affiliations:** Department of Experimental Medicine, Faculty of Medicine, University of Lleida, Lleida, Spain; Medical Clinic, University Hospital Tuebingen, GERMANY

## Abstract

Soybean is recognized as rich source of bioactive compounds for the improvement of glucose homeostasis. However, the post-prandial mechanisms of action have not been extensively described. The aim of this study is to determine the changes in glucose homeostasis and related factors after acute intake of a soy beverage. Twenty-nine subjects (15 women and 14 men, with an average age of 19.5 ± 1.2) ingested 500 mL of water, glucose (20.5 g/500 mL) and soy beverage (20 g of carbohydrate) in three separate sessions. Capillary blood glucose was monitored every 15 min until 120 min post-prandial, and blood samples were collected at baseline and after 60 min for insulin, incretin, free amino acids, antioxidant capacity and inflammation marker analysis. The increase in capillary glucose after soy-beverage intake was negligible. This is explained in part by an increase in 83% in insulin levels than induced with glucose alone, which is mainly mediated by a low insulin degradation ratio (determined by c-peptide ratio), incretins and likely also by the modulation of the antioxidant environment. No associations were observed between the insulin levels and soy amino acid uptake. It could be concluded that the acute low glycaemic response of a soy beverage may involves a relationship between incretin and insulin secretion and insulin degradation.

## Introduction

Over the last decades, scientific evidence of the impact of soy foods and supplements has grown considerably, and in most countries there is broad social acceptance of their health benefits and, in some cases, therapeutic role in glucose and insulin management. Soybean is a rich source of vegetable proteins, complex carbohydrates, polyunsaturated fat, soluble fibres and isoflavones that may be beneficial in the prevention of insulin resistance. With regard to the effects of soy foods on glucose homeostasis, several studies have shown that soy products and foods significantly improve glycaemic control [1]. *In vitro* studies suggest that the α-glycosidase and tyrosine kinase inhibitory properties of soy isoflavones may inhibit intestinal glucose uptake [2,3]. Human studies have indicated that soy protein or isoflavones may improve glycaemic control and increase insulin sensitivity, lowering the risk of diabetes [4,5]

Despite these findings, it is unclear whether the effect of soy products on glycaemic homeostasis is associated with changes in insulin secretion or peripheral insulin sensitivity. Insulin secretion by pancreatic β-cells is mainly regulated by plasma glucose concentrations. However, other hormones like GIP and GLP-1, nutrients such as free fatty acids and amino acids, and bioactive compounds may also be involved in this process [6]. For example, some amino acids such as arginine, alanine, glutamine and glycine may modify the membrane potential, opening the voltage-sensitive calcium channels and increasing insulin secretion [7]. Interestingly, the isoflavones genistein and daidzein also modify insulin secretion mediated at least in part by the cAMP-dependent protein kinase pathway [8]. Furthermore, genistein and daidzein have been shown to stimulate GLP-1 secretion in enteroendocrine NCI-H716 cells [9], although the phytoestrogen effect on insulin secretion has not been demonstrated *in vivo*.

Overall, the post-prandial glycaemic response to soy product intake is known to be lower than that of other high-carbohydrate foodstuffs. However, it is not clear whether the low glycaemic response is due to low carbohydrate bioavailability or to the effects of soy foods on insulin secretion or sensitivity. Therefore, the objective of this study was to evaluate glycaemic response, insulin secretion and other related factors after soy-based beverage intake in healthy subjects. To achieve this, a cross-over study was performed to determine the effects of the soy beverage in glucose homeostasis relative to the changes induced by a glucose load and by water as a control.

## Materials and methods

### Study population and study design

A cross-over study design was implemented in which each volunteer was given in three separate and non-consecutive sessions either a 500 mL intake of water, or 500 mL glucose solution (20.5 g/500 mL), or 500 mL of a soy beverage. Twenty-nine non-smokers (15 women and 14 men with an average age of 19.5 ± 1.2) from Lleida (Catalonia, Spain) were recruited through brochure distribution within the University community and via the research group’s website during the months of February-March 2015. Inclusion criteria were the following: a) no known food allergy or intolerance to soy or soy products; b) no medication use known to affect glucose tolerance; whereas exclusion criteria included: a) a known history of diabetes; b) a major medical or surgical event requiring hospitalization within the preceding 3 months; c) the presence of disease or drug which influence digestion and absorption of nutrient; d) the use of steroids, protease inhibitors or antipsychotics. Baseline demographic characteristics of the study population are described in S1 Table. Subjects gave their written informed consent to participate in the intervention, and all experimental procedures were reviewed and approved by the Ethical Committee of the Hospital Arnau de Villanova (CEIC-1167); and complied with the ethical standards laid down in the policy statements of the University of Lleida Institutional review board. Baseline characteristics were obtained, and no statistical differences in anthropometrics and biochemical parameters were found between subjects. Subjects were allowed to follow their regular diet the day before the analysis. On the day of the analysis, all subjects were at least 8 h-fasted before the analysis, which took place at 8:00 a.m. The experimental procedure consisted of a baseline blood sample collection followed by the intake of either 500 mL of water, or 500 mL of a glucose solution (20.5 g in 500 mL) or 500 mL of a soy beverage in less than 10 minutes, and a further blood sample collection at 60 min post-prandially. The sequence of the treatments where randomized between the subjects and one week of wash-out period was left between the days of analysis. To monitor the post-prandial glycaemic response, capillary blood samples were taken every 15 min until 120 minutes and blood glucose levels were determined using an ACCU-CHEK Aviva blood glucose glucometer (Roche Diagnostics Gmbh, Mannheim, Germany).

### Beverage description

The soy beverage used in the study is commercially available in Spain (Grupo Leche Pascual, Aranda de Duero, Spain). The nutritional composition is shown in Table 1, which reproduces the nutritional information indicated on the product packaging. The beverage is fortified with vitamin A & D (retinol and 25(OH) hydroxycholecalciferol, respectively).

**Table 1:** Soy beverage nutritional composition per 100 mL. The beverage is commercially available in Spain and has a soy content of 13% and added vitamins A & D and calcium.

Nutrient	Content per 100 mL
Energy (kJ)	185
Protein (g)	3.1
Carbohydrates (g)	4.1
Sugars (g)	2.7
Fibre (g)	0.5
Lipids (g)	1.7
Saturated fat (g)	0.26
Monounsaturated fat (g)	0.40
Polyunsaturated fat (g)	1.04
Omega 6 (g)	0.9
Omega 3 (g)	0.14
Vitamin A (Retinol) (μg)	120
Vitamin D (25(OH)D3) (μg)	0.75
Calcium (mg)	120

### Blood biochemical analyses

Blood samples were collected by venipunction into Vaccutainer® tubes. LDL cholesterol, HDL cholesterol, triglycerides, uric acid and lactate were determined by the Biochemical Analysis Services of the Hospital Universitari Arnau de Vilanova (Lleida, Spain). Insulin, GIP, GLP-1, TNFα, Leptin and IL-6 were determined using a xMAP Milliplex kit (Human Metabolic Hormone Panel Cat. #HMH-34K Millipore) following manufacturer’s instructions. C-peptide was determined by ELISA (Millipore EZHCP-20K) following manufacturer’s instructions. Antioxidant capacity was measured by the FRAP assay following the procedure described by Benzie & Strain [10], using Trolox as antioxidant capacity standard. Dipeptidil peptidase IV activity was measured following the procedure described by Christopher et al [11].

### Free amino acid plasma concentrations

Free plasma amino acids were determined by HPLC-UV with the Waters AccQ.Tag Chemistry Package (WAT052875, Waters, United States). Free amino acids were extracted from plasma after protein precipitation with 5% perchloric acid (50:50 plasma: 5% perchloric acid). Samples were centrifuged at 15,000 rpm for 10 min at 4°C. The supernatant was collected and AccQ.Flour Reagent was used to derivatize the amino acids. Derivatives were separated by reversed-phase HPLC and quantified by UV detection at 254 nm following manufacturer’s instructions.

### Statistics

Statistical analysis was conducted in SPSS 17.0 for Windows. Normality of variables was checked using the Kolmogorov-Smirnov test. One-way ANOVA or the paired Student t-test was performed within each of the groups for the different parameters determined, with post-hoc analyses when appropriate. Differences of p < 0.05 were considered statistically significant. Data are presented as mean ± standard deviation for the number of subjects in each group. The area under the curve was calculated by the trapezoidal method. Individual data points are included in S1 Data.

## Results

**Fig 1:**
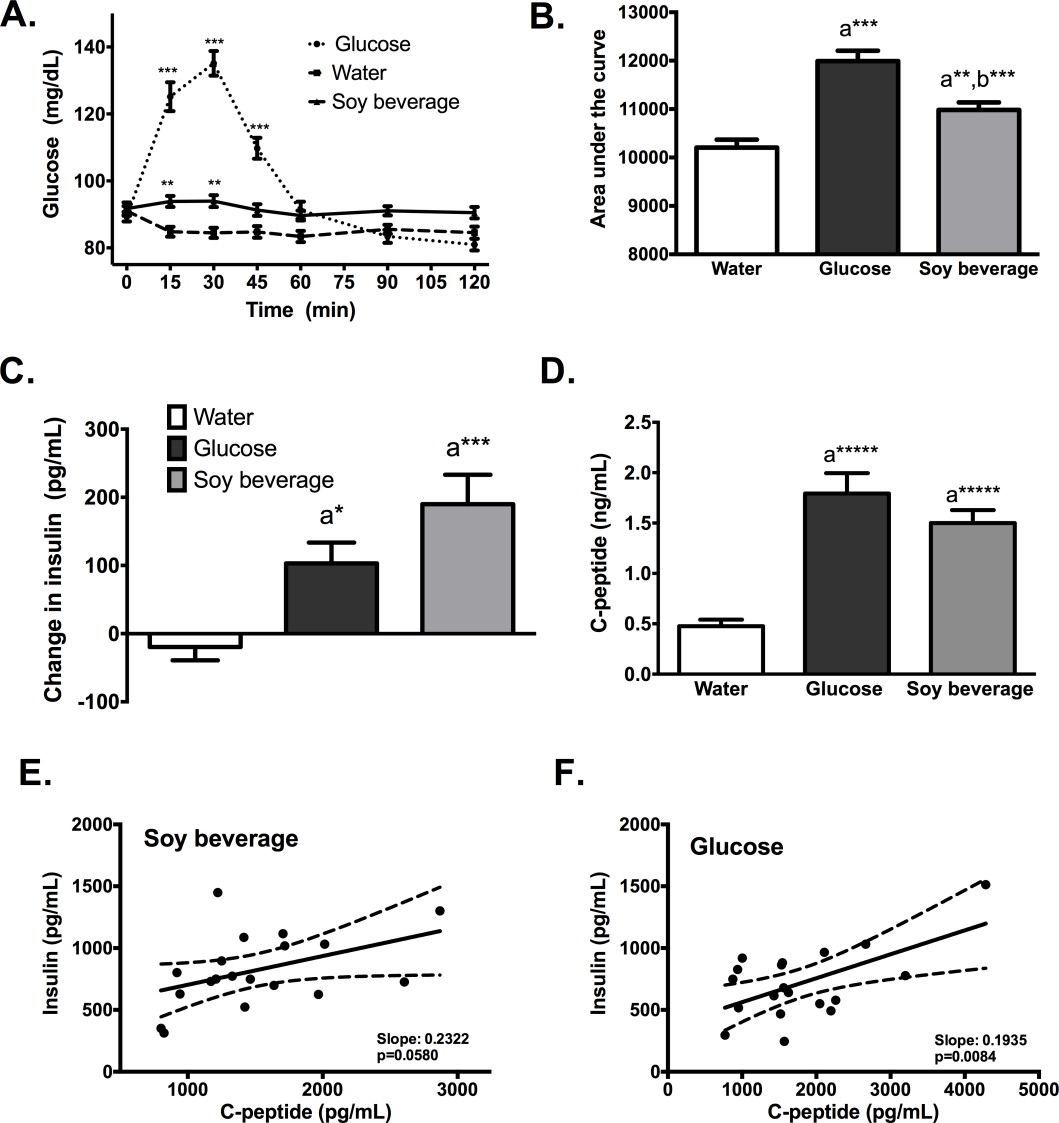
Post-prandial glycaemic response to soy-based beverage consumption. (A) Capillary glucose levels after the ingestion of 20.5 g of glycaemic carbohydrates of a soy-based beverage (500 mL), 20.5 g of glucose in 500 mL of water, and 500 mL of water. (B) Area under the curve for increases in glycaemia. (C) Changes in plasma insulin content 60 min after ingestion of the soy beverage, glucose or water. (D) Blood c-peptide content 60 min after ingestion of the soy beverage, glucose or water. Correlation between blood insulin levels and c-peptide after soy beverage intake (E) and glucose (F). The data are expressed as the difference between basal conditions and insulin content at 60 min post-prandial. Statistically significant differences are denoted by * (p < 0.05); ** (p < 0.01); *** (p < 0.001); a and b denote statistically significant differences from the water and glucose groups respectively. One-way ANOVA analysis was performed to identify the differences between the groups.

**Fig 2:**
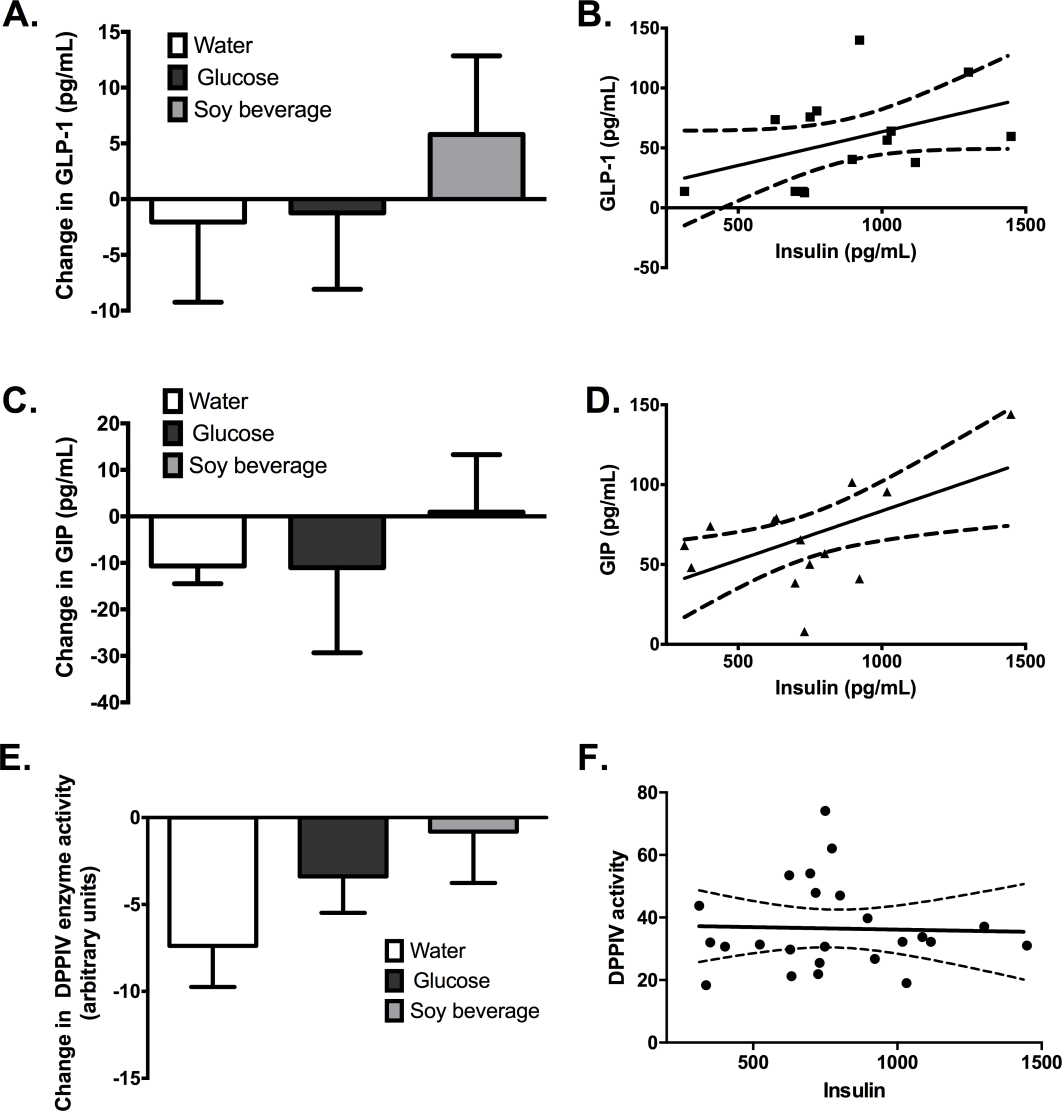
Changes in incretin levels 60 min after ingestion of the soy-based beverage and the corresponding controls (glucose and water); correlation with insulin levels. (A), (C) and (E) show the differences in GLP-1 and GIP concentrations and DPPIV enzyme activity 60 min after ingestion of the test compound. Paired t-test paired analysis was performed to identify the differences in concentration of each parameter; no differences were observed in GLP-1, GIP or DPPIV (p values = 0.6915, 0.7513 and 0.8778, respectively). Pearson correlation was observed between the levels of insulin (60 min. post-prandial) and GLP-1 and GIP (p values = 0.0096 and 0.0410, respectively); no correlation was observed with DPPIV enzyme activity. (B); (D) and (F) show the estimated linear regression between the levels of insulin and GLP-1, GIP and DPPIV enzyme activity. GIP and insulin levels showed significant Pearson correlation coefficients (p = 0.0410).

The soy beverage tested in this study performed an estimated glycaemic index of 43% (compared to the glucose load) (Fig 1), making it a low glycaemic index food. The low index is in response to the negligible increase in post-prandial glucose levels (Fig 1(A); e.g., an increase of 2 ± 8 mg/dL at minute 15 and 2±10 mg/dL at minute 30). This behavior suggest that the effects of the soy beverage on plasma glucose levels are rapidly triggered and may be due, in part, to the reduction of carbohydrate absorption in the gut and, principally, to the increased levels of insulin (Fig 1(C)), which was 83% higher than induced by glucose alone. Similar post-prandial values of c-peptide were observed after soy beverage and glucose intake (Fig 2(D)). Notwithstanding, the ratio between post-prandial insulin and c-peptide levels was different between the two treatments. For instance, the insulin/c-peptide ratio at 60 min post-prandial was 0.2322 and 0.1935 for the soy beverage and glucose respectively (Fig 1(E) and 1(F)).

Secretagogue effects in pancreatic β-cells have been attributed to certain amino acids like glutamine, phenylalanine and leucine [12]. Consequently, to determine if the increase in post-prandial blood concentrations of amino acids could be related to the increase in insulin levels, the plasma concentration of each amino acid was determined (S2 Table). In general terms, 60 min after glucose solution and water intake, a reduction in the plasma content of almost all amino acids was observed. By contrast, after soy beverage intake, a significant increase in serine, histidine, arginine, threonine, proline, valine, lysine and leucine was observed, with increases ranging from 13% for histidine to 33% in the case of leucine. No significant correlation was observed between the plasma levels of each amino acid and insulin, but the sum of amino acids showed a significant direct correlation (p = 0.0012) with insulin levels at 60 min post-prandial, with a Pearson coefficient of 0.5713, suggesting that the observed effect may not be due to an unique, direct interaction between individual amino acid levels and insulin secretion.

Despite these findings, the increase in plasma amino acids only accounts for part of the observed post-prandial insulin levels after the soy beverage; other gut hormonal factors such as incretin secretion have also been suggested to contribute to the increase in insulin levels. Glucose-dependent insulinotropic polypeptide (GIP) and glucagon-like peptide-1 (GLP-1) are intestinal hormones secreted in response to the ingestion of various nutrients [13]. These incretins stimulate insulin secretion from pancreatic β-cells in a glucose-dependent fashion. Fat and carbohydrate intake are the major stimuli for GIP and carbohydrates for GLP-1. Compared to the ingestion of water or the glucose solution, the soy beverage did not induce a significant increase in the levels of GIP and GLP-1 (Fig 2(A) and 2(C)). However, there was a significant direct correlation between 60 min post-prandial levels of GIP and GLP-1 and insulin (Fig 2(B) and 2(D)) after soy beverage intake; correlation not observed for glucose and water. Soy or soy compounds may induce changes in incretin levels through direct stimulation of K- or L-cells, or by inhibiting dipeptydil peptidase-4 (DPPIV) activity. In this context, to elucidate the causes of the increase in incretin levels after soy beverage intake, the activity of DPPIV was determined (Fig 2(E)). At 60 min post-prandial, DPPIV activity was found to be higher after water consumption (p < 0.0001) than after intake of the glucose solution or the soy beverage, and no differences were observed between the effects of glucose or soy intake (p = 0.5263). In addition, no correlation was found between post-prandial DPPIV activity and insulin levels (Fig 2(F)), which suggests that the increased levels of GIP and GLP-1 after soy beverage intake may not be the result of inhibited DPPIV activity.

With regard to other factors that may stimulate insulin secretion, lipid receptors in pancreatin β-cells (e.g., GPR40, GPR119) may increase circulating insulin levels through a direct insulinotropic action on β-cells and through stimulation of incretin secretion [14]. In fact, a reduced blood lipid profile was observed after glucose solution intake, whereas no differences were observed after soy beverage intake (Table 2).

**Table 2:** Changes observed between basal conditions and 60 min post-prandial. The paired Student t-test was performed in each group for the parameters analysed. Statistically significant differences induced by intake are shown in bold.

	Basal	Post-prandial	p	p value for intervention-derived differences^1^
**A. Soy beverage (500 mL)**				
Lipid profile (mg/dL)				
LDL	**92 ± 22**	**82 ± 24**	0.0151	0.2652
HDL	53 **±** 12	53 **±** 15	0.8145	0.1306
Triglycerides	92 **±** 34	95 **±** 42	0.5712	0.0131
Inflammation biomarkers (pg/mL)				
TNFα	**9.8 ± 3.6**	**8.9 ± 4.1**	0.0067	0.3166
IL-6	67 **±** 63	63 **±** 57	0.1336	0.5551
Other				
FRAP (microM TE)	**596 ± 131**	**613 ± 121**	0.0380	0.0001
Uric acid (mg/dL)	**4.7 ± 1.6**	**5.0 ± 1.5**	0.0004	0.0001
Leptin (pg/mL)	**8815 ± 7050**	**7408 ± 6598**	< 0.0001	0.1355
Lactate (mg/dL)	17 **±** 6	16 **±** 4	0.0729	0.4644
**B. Glucose (20.5 g in 500 mL)**				
Lipid profile (mg/dL)				
LDL	**97 ± 27**	**91 ± 30**	0.0037	0.2652
HDL	58 **±** 13	57 **±** 12	0.3577	0.1306
Triglycerides	**105 ± 44**	**92 ± 49**	0.0010	0.0131
Inflammation biomarkers (pg/mL)				
TNFα	**11.2 ± 4.5**	**9.5 ± 3.8**	0.0058	0.3166
IL-6	55 **±** 35	51 **±** 40	0.4170	0.5551
Other				
FRAP (microM TE)	**551 ± 118**	**526 ± 113**	< 0.0001	0.0001
Uric acid (mg/dL)	4.2 **±** 1.5	4.0 **±** 1.3	0.0807	0.0001
Leptin (pg/mL)	**8409 ± 8357**	**7202 ± 8075**	0.0003	0.1355
Lactate (mg/dL)	23 **±** 13	19 **±** 6	0.4452	0.4644
**C. Water (500 mL)**				
Lipid profile (mg/dL)				
LDL	88 **±** 19	85 **±** 24	0.2797	0.2652
HDL	51 **±** 12	54 **±** 13	0.1080	0.1306
Triglycerides	98 **±** 41	90 **±** 52	0.0997	0.0131
Inflammation biomarkers (pg/mL)				
TNFα	10.6 **±** 3.6	10.0 **±** 3.6	0.1155	0.3166
IL-6	72 **±** 60	71 **±** 64	0.9187	0.5551
Other				
FRAP (microM TE)	**566 ± 119**	**542 ± 108**	0.0132	0.0001
Uric acid (mg/dL)	**4.8 ± 1.6**	**4.6 ± 1.6**	0.0376	0.0001
Leptin (pg/mL)	**9886 ± 8196**	**7408 ± 6598**	< 0.0001	0.1355
Lactate (mg/dL)	**17 ± 6**	**12 ± 4**	<0.0001	0.4644

^1^Evaluated by ANOVA on the paired differences.

**Fig 3:**
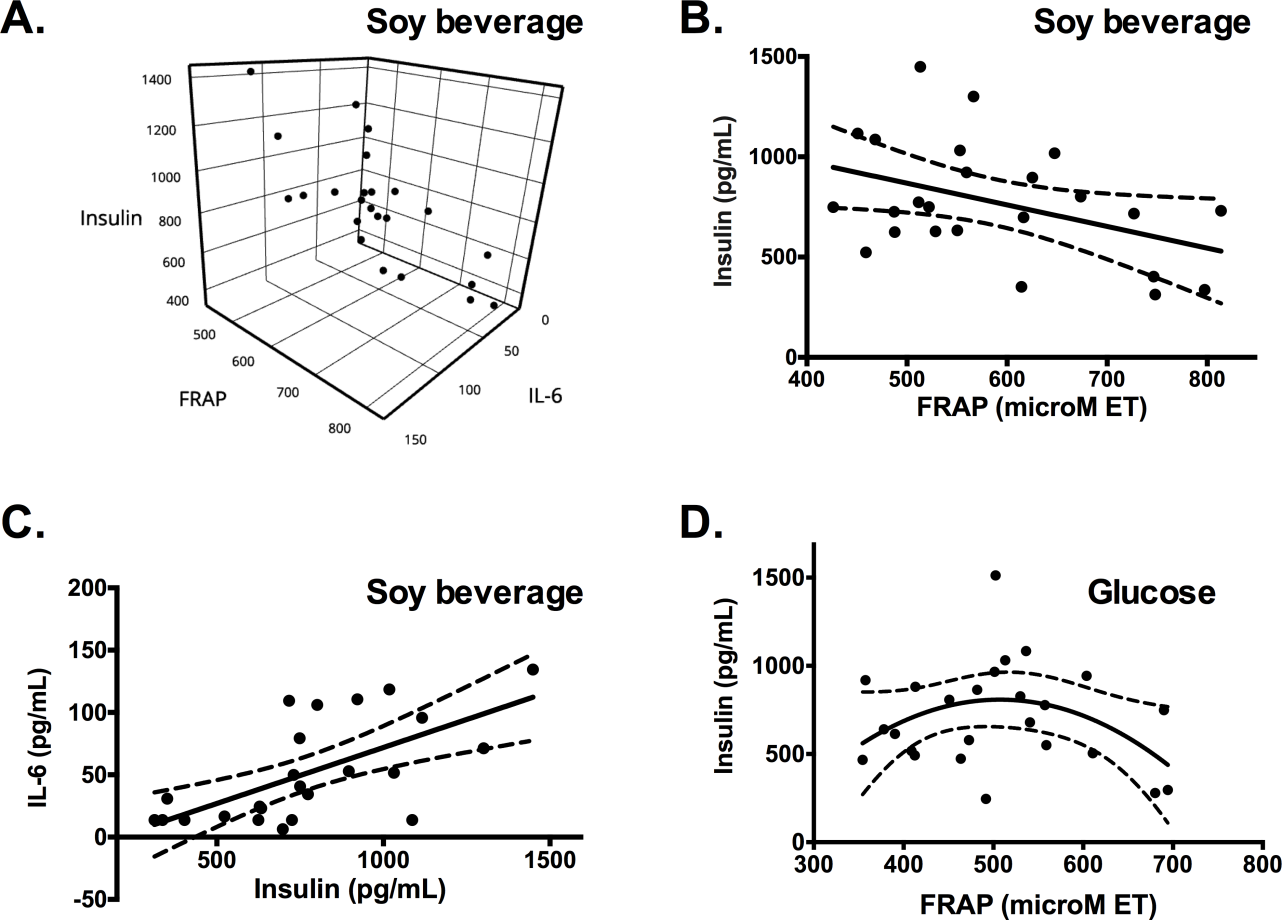
Correlation between post-prandial plasma levels of insulin, IL-6 and antioxidant capacity measured by FRAP. (A) Estimated predictive model of insulin levels relative to IL-6 and antioxidant capacity, following the equation: Insulin (pg/mL) = 4.52 (IL-6 pg/mL) -1.03 (FRAP μM TE) + 1146.8. The observed potency is 0.988 and 0.766 for IL-6 and FRAP, respectively. Correlation between insulin levels and antioxidant capacity (B) and IL-6 (C) after soy beverage intake. Pearson correlation was observed for antioxidant capacity p = 0.0007 and IL-6 p = 0.0416. (d) “∩” behaviour observed between plasma antioxidant capacity measured by FRAP and insulin content after glucose load.

Other blood parameters, shown in Table 2, could be also related to the increased insulin levels recorded after soy beverage intake. For example, a significant increase in plasma antioxidant capacity measured by the FRAP assay was observed. Similarly, the levels of antioxidant capacity were only correlated with insulin levels after soy beverage intake (Pearson correlation p = 0.0416); however, an inverse correlation was observed, with higher FRAP levels linked to lower plasma insulin concentration (Fig 3(B)). On the basis of this behaviour, the effect of plasma antioxidant capacity after glucose intake was evaluated (Fig 3(D)). It was observed that the antioxidant capacity might induce a non-linear “∩”-shape relationship (determined by a second order polynomial regresion p = 0.2632) on glucose-stimulated insulin secretion, in which at low and high antioxidant capacities the release of insulin by β-cells is reduced, whereas at intermediate capacities the insulin levels are higher. In addition, in this study it was found that IL-6 was directly correlated with insulin (p = 0.0007 for the soy beverage, p = 0.0015 for the glucose solution and p = 0.0001 for water). Similarly, it was found that the antioxidant capacity measured by FRAP and IL-6 could predict insulin levels (Fig 3(A)), whereas at higher levels of IL-6 and intermediate antioxidant capacity the insulin levels were higher.

## Discussion

Tight glycaemic control is necessary to maintain health and to prevent disease. Post-prandial hyperglycaemia and related hyperinsulinemia and hyperlipidaemia have been implicated in the aetiology of chronic metabolic diseases such as type 2 diabetes mellitus and cardiovascular disease. Additionally, there is evidence that post-prandial glycaemia may affect body weight control by altering the balance between fat and carbohydrate oxidation, thereby influencing energy expenditure.

Since the concept of glycaemic index was introduced in 1981 [15], several studies have been undertaken to elucidate the types of foods that may induce lower post-prandial glycaemia. The relationship between food and blood hyperglycaemia is know to be influenced mainly by carbohydrate quality and other food-related compounds rather than by carbohydrate quantity *per se*. Soy and soy products are generally acknowledged as inducing a reduced glycaemic response, ranging from 18% for cooked soybeans to 30–44% for soybean beverages [16]. Several soy compounds have been suggested to account for the reduced response of post-prandial glucose levels; soy protein, isoflavones and fibre have been reported to replicate the low glycaemic behaviour observed [4,5]. However, the mechanism of action is not altogether clear, since most of the compounds studied can present different degrees of bioavailability that suggest different active sites for each soy bioactive compound. In this context, the soy beverage tested in this study induces low post-prandial glycaemia that could be explained by three main mechanisms: 1) a reduction in the absorption of digestible carbohydrates; 2) an increase in insulin secretion or a reduction in its degradation; and 3) systemic uptake of the absorbed glucose.

The results of this study demonstrate that the soy beverage may, to a considerable extent, regulate post-prandial glycaemia through an acute increase in insulin levels. This effect may be related to a reduction in insulin clearance, to the higher levels of post-prandial incretins *per se*, or to the increase in antioxidant capacity, which has been suggested to increase insulin sensitivity and glucose-stimulated insulin secretion and to decrease inflammation.

The acute effect of soybean compounds on incretin secretion has not been widely studied. Only few studies have reported an increase in GLP-1 and GIP after the ingestion of soy foods [17]. Some of the available data suggest that the intake of specific amino acids (leucine, isoleucine, valine, lysine and threonine) together with glucose reduces the post-prandial glycaemic response [18] and that this effect is correlated with the increase in GIP and GLP-1. In this sense, a positive effect of leucine and phenylalanine on insulin secretion was reported in a clinical assessment of type II diabetes patients and controls, which showed in a 3-fold increase in secretion compared with carbohydrate alone [12]. In the present study, a clear increase in soy-derived amino acids (serine, histidine, arginine, threonine, proline, valine, lysine and leucine) was observed, but there was no direct correlation between the increase in specific amino acids and the post-prandial levels of insulin. Similar findings were described previously [19] during hyperglycemic clamps, where soy protein stimulated insulin secretion in a lower extent.

With regard to oxidative status, it has been observed that plasma antioxidant capacity is significantly reduced during the oral glucose tolerance test, in which plasma levels of thiol groups, ascorbic acid, tocopherols and uric acid are also reduced [20]. Supporting the hypothesis that oxidative stress may be a significant factor in glucose intolerance, data from studies conducted in cell culture systems indicate that products of oxidative stress impair insulin-mediated translocation of GLUT4 in myotubes and adypocytes [21–23] and suppress gene transcription of adiponectin in adipocytes [24] and insulin in pancreatic β-cells [25]. In β-cells, following glucose uptake phosphorylation, glucose oxidation, which involves both cytosolic and mitochondrial processes, generate signals that trigger insulin secretion [26]. In these types of processes, reactive oxygen species (ROS) derived from glucose metabolism may serve as metabolic signals for glucose-stimulated insulin secretion [27], where an imbalance between ROS formation and removal may lead to an excessive or low concentration of ROS that can lead to the inhibition of glycolysis and the tricarboxylic acid cycle, reducing stimulus-secretion coupling factor generation and impairing insulin secretion [28]. Therefore, the elevation of ROS above a critical threshold may lead to the activation of oxidative stress concomitantly with reduced insulin secretion.

The results of this study suggest that oxidative status may also play an important role in pancreatic β-cell insulin secretion and that the redox status might have a “∩”-shape effect on insulin secretion. At high antioxidant status, the glycolysis-derived ROS needed to stimulate insulin secretion could be down-regulated, whereas at low antioxidant capacity and in the presence of high levels of ROS, β-cell glycaemic metabolism and, therefore, insulin secretion capacity may be reduced. The data from this study show that the plasma antioxidant capacity was maintained after soy beverage intake, which may result in adequate antioxidant status that could promote insulin secretion.

Other soybean-derived antioxidant compounds like isoflavones have also been demonstrated to improve glucose homeostasis. For instance, genistein and daidzein have been shown to prevent diabetes onset in non-obese diabetic mice by preserving pancreatic β-cell function [29]. In the same study, total pancreatic insulin content was 2.5-fold higher in soy-fed mice, despite comparable pancreas weight and similar levels of pancreatic glucagon. Additionally, glucose-stimulated insulin secretion was 3 times greater in soy-fed mice than in those fed the control diet. The improvement of insulin secretion by isoflavones has also been described in *in vitro* assays and could be associated with the insulin/insulin-like growth factor-1 signalling that is activated by tyrosine phosphorylation of insulin receptor substrate 2 and serine phosphorylation of Akt, which, in turn, are increased by pancreatic-duodenal homeobox-1 expression [9].

Limitations of this study may include several questions: 1) the lack of a control beverage that resembles the exact composition of protein, carbohydrate, lipid and bioactive compounds content of the soy beverage, which may be required to specifically dissect the individual effects of these bioactive nutrients. However, accounting the potential number of combinations, this would be largely unpractical; ii) the average age of participants as well as its health state do not allow us to extrapolate the presented results to insulin resistant individuals, as we do not have included a group with higher age or with insulin resistance (or even obese individuals). Though this limitation, the study could be easily extended to these clear targets of treatment with soy-based products in search of a better glycemic status; iii) the number of samples obtained after each beverage intake do not allow us to ascertain a more accurate view of time-dependent postprandial changes in the factors evaluated; as above, though, focusing on healthy individuals can help to characterize the response–in terms of glycemic index- and its physiological determinants; iv) the lack of an exhaustive analyses for all the incretin molecules, as well as the fact that it was not tested the proposed mechanisms in an *in vitro* (e.g. cell culture) or *in vivo* preclinical model (e.g. transgenic model) does not allow to discard other mechanisms of actuation of the soy-beverage. However, based on the published effects of soy components, we think that the offered hypotheses could be of usefulness in explaining the effects a low-glycemic index component of diets.

## Conclusions

Taken together, this evidence suggests that the low glycaemic response of soy foods could be attributable to their insulinotropic effect. The mechanisms of action could be attributed to the stimulation of enteral incretin secretion and antioxidant environment regulation, both of which may stimulate insulin release.

## Supporting information

S1 Table Baseline demographic characteristics of the study population. (DOCX)

S2 Table Changes in serum free amino acid profiles at baseline and after 60 min of test initiation. (DOCX)

S1 Data Individual data points. (XLSX)
