# Supplementary material for: Characterization of the post-prandial insulinemic response and low glycaemic index of a soy beverage
Source: PLoS One. 2017 Aug 9;12(8):e0182762. doi: 10.1371/journal.pone.0182762 (PMC5549974; doi:10.1371/journal.pone.0182762)
Supplement: S1 Table — (DOCX) [file pone.0182762.s001.docx]

**S1 Table.** Baseline demographic characteristics of the study population.

| Parameter | Male | Female |
| --- | --- | --- |
| Number of volunteers | 14 | 15 |
| Age (years) | 19.0 ± 0.4 | 20.0 ± 1.4 |
| Body mass index (kg/m^2^) | 22.4 ± 3.1 | 21.2 ± 1.6 |
