# Supplementary material for: Characterization of the post-prandial insulinemic response and low glycaemic index of a soy beverage
Source: PLoS One. 2017 Aug 9;12(8):e0182762. doi: 10.1371/journal.pone.0182762 (PMC5549974; doi:10.1371/journal.pone.0182762)
Supplement: S2 Table — (DOCX) [file pone.0182762.s002.docx]

**S2 Table.** Changes in serum free amino acid profiles at baseline and after 60 min of test initiation. The results are expressed as mean ± standard deviation of the mM concentrations of each amino acid in plasma. Paired t-test analysis was performed to identify the differences between basal and post-prandial conditions. Values in bold highlight statistically significant differences (p < 0.05) between basal and post-prandial conditions.

|  |  | Water | | | Glucose | | | Soy beverage | | |
| --- | --- | --- | --- | --- | --- | --- | --- | --- | --- | --- |
|  | Amino acid | Basal | 60 min | *p* | Basal | 60 min | *p* | Basal | 60 min | *p* |
| All cases | SER | 101 ± 22 | 100 ± 25 | *0.8938* | 101 ± 20 | 91 ± 21 | *0.1096* | **98 ± 27** | **122 ± 28** | ***0.0031*** |
|  | GLU | 80 ± 37 | 83 ± 44 | *0.8200* | **55 ± 16** | **42 ± 10** | ***0.0273*** | 54 ± 20 | 47 ± 8 | *0.4207* |
|  | GLY | 138 ± 48 | 120 ± 30 | *0.1309* | 125 ± 26 | 131 ± 59 | *0.2635* | 151 ± 87 | 165 ± 108 | *0.0855* |
|  | HIS | 322 ± 95 | 307 ± 83 | *0.1851* | 369 ± 85 | 365 ± 82 | *0.5497* | **372 ± 88** | **421 ± 129** | ***<0.0001*** |
|  | ARG | 65 ± 40 | 54 ± 16 | *0.3462* | 65 ± 26 | 57 ± 25 | *0.1416* | **55 ± 30** | **77 ± 31** | ***0.0043*** |
|  | THR | 76 ± 32 | 73 ± 24 | *0.7943* | 72 ± 33 | 69 ± 32 | *0.7291* | **75 ± 29** | **93 ± 32** | ***0.0252*** |
|  | PRO | 76 ± 28 | 73 ± 22 | *0.7908* | 77 ± 21 | 70 ± 20 | *0.2706* | **73 ± 18** | **90 ± 25** | ***0.0308*** |
|  | TYR | 38 ± 13 | 34 ± 10 | *0.7543* | 42 ± 9 | 35 ± 7 | *0.2189* | 42 ± 9 | 44 ± 9 | *0.8244* |
|  | VAL | 135 ± 44 | 125 ± 36 | *0.4183* | **136 ± 35** | **117 ± 32** | ***0.0010*** | **137 ± 33** | **157 ± 34** | ***0.0095*** |
|  | MET | 28 ± 18 | 24 ± 6 | *0.7584* | 25 ± 5 | 22 ± 6 | *0.5669* | 24 ± 5 | 27 ± 6 | *0.6721* |
|  | LYS | 106 ± 30 | 105 ± 35 | *0.9036* | **105 ± 26** | **93 ± 28** | ***0.0459*** | **101 ± 30** | **131 ± 40** | ***0.0002*** |
|  | ILEU | 42 ± 9 | 40 ± 9 | *0.8409* | 42 ± 9 | 34 ± 8 | *0.1465* | 42 ± 9 | 54 ± 14 | *0.1172* |
|  | LEU | 64 ± 18 | 58 ± 18 | *0,6220* | **64 ± 17** | **46 ± 15** | ***0.0031*** | **61 ± 15** | **80 ± 22** | ***0.0154*** |
|  | PHE | 35 ± 6 | 32 ± 6 | *0,8201* | 34 ± 4 | 29 ± 5 | *0.3405* | 32 ± 6 | 41 ± 7 | *0.2635* |
|  |  |  |  |  |  |  |  |  |  |  |
| Men | SER | 110 ± 29 | 112 ± 32 | *0.9005* | 107 ± 24 | 104 ± 24 | *0.4502* | 101 ± 30 | 120 ± 30 | *0.0676* |
|  | GLU | 82 ± 57 | 119 ± 33 | *0.8313* | 63 ± 16 | 48 ± 10 | *0.2969* | 73 ± 30 | 46 ± 3 | *0.6210* |
|  | GLY | 143 ± 32 | 129 ± 32 | *0.1566* | 133 ± 24 | 127 ± 18 | *0.5928* | 125 ± 33 | 139 ± 36 | *0.2902* |
|  | HIS | 352 ± 73 | 266 ± 62 | *0.2141* | 383 ± 70 | 377 ± 68 | *0.7780* | **341 ± 82** | **438 ± 112** | ***0.0001*** |
|  | ARG | 65 ± 44 | 47 ± 13 | *0.3774* | 53 ± 14 | 60 ± 33 | *0.4880* | 45 ± 18 | 69 ± 26 | *0.0784* |
|  | THR | 58 ± 16 | 65 ± 16 | *0.8071* | 59 ± 10 | 53 ± 16 | *0.8703* | 56 ± 9 | 76 ± 18 | *0.1680* |
|  | PRO | 86 ± 21 | 77 ± 16 | *0.8038* | 86 ± 19 | 78 ± 16 | *0.6034* | 77 ± 20 | 94 ± 26 | *0.1835* |
|  | TYR | 47 ± 9 | 35 ± 8 | *0.7695* | 48 ± 9 | 39 ± 6 | *0.5620* | 43 ± 11 | 46 ± 10 | *0.8917* |
|  | VAL | 177 ± 34 | 151 ± 39 | *0.4483* | 161 ± 36 | 141 ± 30 | *0.1195* | 156 ± 35 | 176 ± 38 | *0.1097* |
|  | MET | 28 ± 5 | 28 ± 5 | *0.7733* | 26 ± 4 | 24 ± 3 | *0.7873* | 26 ± 3 | 26 ± 5 | *0.7950* |
|  | LYS | 128 ± 38 | 124 ± 41 | *0.9097* | 118 ± 30 | 108 ± 36 | *0.3457* | **112 ± 31** | **142 ± 44** | ***0.0201*** |
|  | ILEU | 50 ± 9 | 47 ± 8 | *0.8508* | 48 ± 8 | 40 ± 7 | *0.4933* | 48 ± 10 | 59 ± 13 | *0.3355* |
|  | LEU | 82 ± 15 | 74 ± 16 | *0.6443* | 75 ± 13 | 61 ± 10 | *0.1611* | 74 ± 17 | 90 ± 21 | *0.1355* |
|  | PHE | 36 ± 4 | 35 ± 6 | *0.8313* | 35 ± 3 | 32 ± 5 | *0.6531* | 32 ± 4 | 40 ± 8 | *0.4921* |
|  |  |  |  |  |  |  |  |  |  |  |
| Women | SER | 98 ± 18 | 92 ± 14 | *0.6933* | 97 ± 15 | 84 ± 15 | *0.4046* | 99 ± 27 | 125 ± 26 | *0.1435* |
|  | GLU | 88 ± 32 | 57 ± 31 | *0.0697* | 50 ± 12 | 38 ± 8 | *0.4854* | 45 ± 11 | 48 ± 10 | *0.8645* |
|  | GLY | 136 ± 57 | 114 ± 28 | *0.1821* | 122 ± 26 | 135 ± 75 | *0.4290* | 170 ± 106 | 184 ± 134 | *0.3161* |
|  | HIS | 296 ± 102 | **337 ± 85** | ***0.0164*** | 360 ± 92 | 358 ± 92 | *0.8817* | 392 ± 85 | 420 ± 139 | *0.1301* |
|  | ARG | 64 ± 36 | 59 ± 17 | *0.7361* | 72 ± 30 | 55 ± 20 | *0.2929* | 63 ± 34 | 82 ± 32 | *0.2324* |
|  | THR | 90 ± 33 | 78 ± 27 | *0.5102* | 79 ± 40 | 80 ± 35 | *0.9431* | 88 ± 30 | 102 ± 34 | *0.3209* |
|  | PRO | 72 ± 32 | 71 ± 26 | *0.9726* | 73 ± 22 | 66 ± 21 | *0.6893* | 73 ± 20 | 89 ± 25 | *0.3317* |
|  | TYR | 31 ± 10 | 33 ± 12 | *0.8785* | 38 ± 8 | 32 ± 6 | *0.7029* | 42 ± 6 | 44 ± 8 | *0.9426* |
|  | VAL | 108 ± 26 | 107 ± 19 | *0.9940* | 120 ± 20 | 101 ± 20 | *0.2373* | 127 ± 31 | 147 ± 26 | *0.2439* |
|  | MET | 28 ± 23 | 22 ± 6 | *0.7330* | 25 ± 5 | 21 ± 6 | *0.7963* | 23 ± 6 | 28 ± 6 | *0.7692* |
|  | LYS | 95 ± 24 | 91 ± 23 | *0.8142* | 97 ± 20 | 83 ± 17 | *0.4020* | 98 ± 31 | 127 ± 36 | *0.1055* |
|  | ILEU | 38 ± 8 | 35 ± 5 | *0.8625* | 38 ± 7 | 29 ± 5 | *0.6057* | 39 ± 7 | 50 ± 13 | *0.4874* |
|  | LEU | 53 ± 13 | 47 ± 10 | *0.7165* | 55 ± 14 | 37 ± 8 | *0.2825* | 55 ± 13 | 73 ± 20 | *0.2688* |
|  | PHE | 33 ± 7 | 30 ± 5 | *0.8232* | 33 ± 5 | 27 ± 4 | *0.6964* | 32 ± 6 | 41 ± 6 | *0.6149* |
